# Comparative Whole-Genome Analysis of *Neisseria gonorrhoeae* Isolates Revealed Changes in the Gonococcal Genetic Island and Specific Genes as a Link to Antimicrobial Resistance

**DOI:** 10.3389/fcimb.2022.831336

**Published:** 2022-02-18

**Authors:** Boris Shaskolskiy, Dmitry Kravtsov, Ilya Kandinov, Sofya Gorshkova, Alexey Kubanov, Victoria Solomka, Dmitry Deryabin, Ekaterina Dementieva, Dmitry Gryadunov

**Affiliations:** ^1^ Center for Precision Genome Editing and Genetic Technologies for Biomedicine, Engelhardt Institute of Molecular Biology, Russian Academy of Sciences, Moscow, Russia; ^2^ State Research Center of Dermatovenerology and Cosmetology, Russian Ministry of Health, Moscow, Russia

**Keywords:** *Neisseria gonorrhoeae*, whole-genome sequencing, NG-MAST, gonococcal genetic island, antimicrobial resistance

## Abstract

Comparative whole-genome analysis was performed for *Neisseria gonorrhoeae* isolates belonging to the *Neisseria gonorrhoeae* multiantigen sequence typing (NG-MAST) types predominant worldwide — 225, 1407, 2400, 2992, and 4186 — and to genogroup 807, the most common genogroup in the Russian Federation. Here, for the first time, the complete genomes of 25 *N. gonorrhoeae* isolates from genogroup 807 were obtained. For NG-MAST types 225, 1407, 2400, 2992, and 4186, genomes from the Pathogenwatch database were used. The phylogenetic network constructed for 150 genomes showed that the clustering according to NG-MAST type corresponded to the clustering according to genome. Comparisons of genomes of the six sequence types revealed 8-20 genes specific to each sequence type, including the loci for phase variations and genetic components of the gonococcal genetic island (GGI). NG-MAST type 2992 and 4186 isolates either lacked the GGI or carried critical mutations in genes essential for DNA secretion. In all analyzed genogroup 807 isolates, substitution of the essential *atlA* gene with the *eppA* gene was found, accompanied by a change in the *traG* allele, replacement of the *ych* gene with *ych1*, and the absence of the *exp1* gene, which is likely to result in loss of GGI functionality. For the NG-MAST type 225, 1407 and 2400 isolates, no premature stop codons or reading frameshifts were found in the genes essential for GGI function. A relationship between isolate susceptibility to ciprofloxacin, penicillin, tetracycline and the presence of lesions in GGI genes necessary for DNA secretion was established. The *N. gonorrhoeae* evolutionary pathways, which allow a particular sequence type to maintain long-term predominance in the population, may include changes in genes responsible for adhesion and virulence, changes in the GGI structure, preservation of genes carrying drug resistance determinants, and changes in genes associated with host adaptation or encoding enzymes of biochemical pathways.

## Introduction

Gonococcal infection caused by *Neisseria gonorrhoeae* is one of the most common sexually transmitted diseases. *N. gonorrhoeae* can quickly acquire resistance to antimicrobial drugs used for the treatment (Unemo and Shafer, 2014; Unemo and Jensen, 2017; Unemo et al., 2019). The development of resistance to third-generation cephalosporins (ceftriaxone and cefixime), the modern drugs of choice for the treatment of gonorrhea worldwide, poses a great danger, since gonorrhea may become an incurable disease (Unemo, 2015; Tacconelli et al., 2018).

The *Neisseria gonorrhoeae* multiantigen sequence typing (NG-MAST) scheme is a traditional tool for studying the molecular epidemiology of gonococcal infection (ECDC report, 2018). This method allows one to perform two related challenging endeavors: first, to isolate a meaningful number of genetic variants [sequence types (STs)] within the *N. gonorrhoeae* species and, on this basis, to analyze the transmission routes of gonococcal infection (Martin et al., 2004); second, to identify and control the spread of the most epidemiologically dangerous clones with multiple resistance to antimicrobial drugs (Chisholm et al., 2013).

New approaches to the study of *N. gonorrhoeae* molecular epidemiology are based on the use of whole-genome sequencing (WGS) technologies, which allow simultaneous evaluation of both the set of genes characterizing the origin and transfer of the analyzed clinical isolate (De Silva et al., 2016) and the set of genetic determinants of antibiotic resistance (Harrison et al., 2016). In fact, WGS allows typing problems to be solved at a higher level than does the NG-MAST technique, directly linking the origin of the analyzed clinical isolates with their antibiotic resistance (Harrison et al., 2020). WGS data have been successfully used to identify the determinants of resistance in multiresistant isolates from Europe (Jacobsson et al., 2016; Abrams and Trees, 2017; Harris et al., 2018; Ryan et al., 2018) and the USA (Grad et al., 2016); to study phylogenetic relationships, population structure and molecular epidemiology (Demczuk et al., 2015; Ezewudo et al., 2015; Grad et al., 2016; Abrams and Trees, 2017; Ryan et al., 2018; Harrison et al., 2020); and to predict the level of resistance to various drugs (Eyre et al., 2017; Golparian et al., 2018).

Horizontal gene transfer is an important driving force of bacterial evolution. The development of genetic diversity can lead to the accumulation of genes and alleles that help bacteria survive by responding to selection pressures, for example, by acquiring antibiotic resistance genes, virulence/pathogenic factors, genes that contribute to evasion of the host immune response, etc. (Dubnau, 1999; Hamilton and Dillard, 2006). For *N. gonorrhoeae*, a key mode of chromosomal DNA transfer is most likely a transformation that occurs frequently and efficiently owing to the natural competence of this bacterium (Hamilton et al., 2005; Hamilton and Dillard, 2006). The type IV secretion system (T4SS) allows the bacterial cell to produce and secrete single-stranded DNA (ssDNA), which can then be specifically recognized by pili on recipient cells *via* DNA uptake sequences (DUS) sequences and recombined into the genome (Hamilton and Dillard, 2006). The T4SS is encoded by genes located on a gonococcal genetic island (GGI) of approximately 59 kb in length (Hamilton et al., 2005; Callaghan et al., 2017; Callaghan et al., 2021). A GGI is present in the genome of ~80% of *N. gonorrhoeae* isolates. Like all genetic islands, it is a mobile element and itself was once acquired by horizontal transfer (Rotman and Seifert, 2014). The site-specific recombination system XerCD is responsible for its mobility, cutting the flanking *difA* and *difB* sites and inserting the island (Dillard and Seifert, 2001; Hamilton et al., 2005; Ramsey et al., 2011; Harrison et al., 2016; Callaghan et al., 2017; Callaghan et al., 2021). As has been shown experimentally, among the 66 GGI genes, only 21 are essential for the function of the system, and 2/3 of the essential genes are *tra* genes, which are homologous to the genes encoding T4SS of the F-plasmid of *E. coli* (Hamilton et al., 2005; Pachulec et al., 2014; Callaghan et al., 2017).

Analysis of the PubMLST (https://pubmlst.org) and Pathogenwatch (https://pathogen.watch/genomes/all?organismId=485) databases showed that the most common STs worldwide are NG-MAST types 225, 1407, 2400, 2992 and 4186. NG-MAST 1407 is predominant in many European countries and causes anxiety due to its multidrug resistance, including resistance to third-generation cephalosporins (ECDC report, 2013; Unemo and Shafer, 2014; Unemo and Jensen, 2017; Unemo et al., 2019; Młynarczyk-Bonikowska et al., 2020). Our phylogenetic analysis of Russian isolates in previous works showed that the Russian population of *N. gonorrhoeae* differs from the European population. Isolates of NG-MAST type 1407 were found only sporadically; isolates of NG-MAST types 225, 2400, 2992, and 4186 were also rare (Kandinov et al., 2020; Shaskolskiy B. et al., 2020; Shaskolskiy B. L. et al., 2020b). The most abundant genogroup in the Russian Federation was the G807 genogroup, accounting for more than 20% of all samples and including the most common NG-MAST types in the Russian Federation, i.e., NG-MAST types 807, 228, 1544, 9570, 9576, and 5941 (Shaskolskiy B. et al., 2020). Notably, ST 807 is rare in Europe: only 5 isolates of this type were identified among 1189 European Centre for Disease Prevention and Control (ECDC) isolates (ECDC report, 2013).

The goal of this work was to perform a comparative whole-genome analysis of *N. gonorrhoeae* isolates of NG-MAST types predominant worldwide and in the Russian Federation that could identify genes and strongly differing alleles specific to each of the STs and identify phylogenetic relationships for the predominant STs. An important part of the work was analysis of the GGI, which is responsible for horizontal gene transfer, and consideration of the relationship between antimicrobial susceptibility and structural changes in the GGI.

## Materials and Methods

### WGS of the Isolates From the Russian Federation

For WGS, 25 previously analyzed clinical *N. gonorrhoeae* isolates were selected (Kubanov et al., 2019; Shaskolskiy et al., 2019; Kandinov et al., 2020) (**Table 1**). The isolates were assigned to ST 807 and to STs 228, 5941, 9570, and 9576 (all belonging to genogroup 807), in which the combined sequences of the *porB* and *tbpB* gene fragments used for NG-MAST typing differed by no more than one nucleotide.

**Table 1:** Clinical isolates of *N. gonorrhoeae* from the Russian Federation used for whole-genome sequencing.

Sample name	Sample code	Year	Region	NG-MAST	Bioproject PRJNA768989 Accession (Sample ID)
1	10500	2018	Astrakhan	9570	SAMN22599447
2	10610	2018	Stavropol	228	SAMN22599455
3	10638	2018	Cheboksary	807	SAMN22600315
4	10531	2018	Arkhangelsk	228	SAMN22599488
5	10524	2018	Arkhangelsk	807	SAMN22599711
6	10562	2018	Kaluga	9576	SAMN22600865
7	10702	2019	Astrakhan	9570	SAMN22600584
8	10727	2019	Arkhangelsk	228	SAMN22600590
9	10794	2019	Cheboksary	9570	SAMN22600605
10	10795	2019	Cheboksary	5941	SAMN22600608
11	10574	2018	Kaluga	9576	SAMN22600672
12	10791	2019	Cheboksary	5941	SAMN22600892
13	10771	2019	Omsk	807	SAMN22600895
14	9399	2016	Omsk	807	SAMN22830696
15	10704	2019	Astrakhan	228	SAMN22830815
16	11000	2016	Kaluga	9576	SAMN22835011
17	9126	2015	Penza	9570	SAMN22835249
18	10269	2017	Kaluga	9576	SAMN22835397
19	10328	2017	Cheboksary	807	SAMN22835445
20	10792	2019	Cheboksary	807	SAMN22835458
21	10231	2017	Astrakhan	807	SAMN22835561
22	10612	2018	Stavropol	228	SAMN22835583
23	10723	2019	Arkhangelsk	807	SAMN22835604
24	10272	2017	Kaluga	807	SAMN22835606
25	10268	2017	Kaluga	9576	SAMN22835617

Isolates were grown aseptically on separate chocolate agar plates at 37°C in the presence of 5% CO_2_. Genomic DNA was isolated from overnight cultures of gonococcal cells using a Monarch Genomic DNA Purification Kit (New England Biolabs, UK). The obtained DNA preparations were additionally purified using AMPure XP magnetic beads (Beckman, USA). Final DNA concentrations were measured using a NanoDrop 2000 spectrophotometer and a Qubit 4 Fluorometer (both Thermo Scientific, USA) and ranged from 20-100 ng/μL.

WGS was performed on two platforms: FLO-MIN110 R9 and R10 flow cells in a MinION device (Oxford Nanopore Technologies, UK) and the MiniSeq system (Illumina, USA). For sequencing in the MinION device, a library of DNA fragments was prepared using Oxford Nanopore and New England Biolabs reagent kits according to the native barcoding genomic DNA protocol (with EXP-NBD104, EXP-NBD114, and SQK-LSK109). According to the manufacturer’s protocol, damaged DNA ends were repaired, and subsequent bar coding and ligation of the adapters were carried out. The final library (5-50 fmol) was loaded onto the flow cell.

DNA libraries for sequencing on the Illumina platform were prepared using the DNA fragmentation method followed by PCR and indexing according to the Nextera XT DNA Library (Illumina) protocol. After purification of the libraries, their sizes and concentrations were checked using an automated TapeStation 4150 capillary electrophoresis platform (Agilent, USA). The final library was normalized and denatured according to the instructions for the MiniSeq instrument. The density of clusters averaged 170-250 K/mm^2^ in all runs. The data output averaged 10.5 gigabytes (GB) out of the 12 GB theoretically possible with this system.

Fifteen *N. gonorrhoeae* isolates were sequenced in parallel on the Oxford Nanopore and Illumina platforms, and 10 isolates were sequenced only on the Illumina platform. In total, ~1 GB of raw data in fastQ format was collected for each sample from each platform. Hybrid assembly of *de novo* genomes after sequencing on two platforms (15 genomes) was carried out using the Unicycler program (https://github.com/rrwick/Unicycler). The genomes of the remaining 10 isolates were assembled using SPAdes v3.13.1 (https://github.com/ablab/spades). Quality trimming and adapter clipping were carried out with Trimmomatic v0.39 (https://github.com/usadellab/Trimmomatic). All sequences were uploaded to GenBank, Bioproject PRJNA768989, under the assigned accession numbers (**Table 1**).

### Selection of *N. gonorrhoeae* Genomes From the Database

To compare genomes and identify genes specific to one ST compared with another, we used the genomes of isolates of the most common STs worldwide available in the Pathogenwatch database (https://pathogen.watch/genomes/all?organismId=485): NG-MAST types 225, 1407, 2400, 2992 and 4186; 25 genomes of each ST; 125 genomes in total. Samples from the database were randomly selected as follows: for each analyzed ST, a list of genomes in the database for the years 2004-2017 was compiled, a set of 25 random unrepeated numbers ranging from 1 to the length of the list was obtained, and genomes were selected in accordance with the set of random numbers. Selected samples are listed in **Table S1** of the **Supplementary Material**.

### Construction of the Phylogenetic Network

The selected genomes of *N. gonorrhoeae* isolates were processed using the Prokka 1.14.6 program (https://github.com/tseemann/prokka) to obtain gff files with annotated genomes. The gff files were then analyzed by rapid large-scale prokaryote pangenome analysis using Roary software (Page et al., 2015). After Roary processing, core genomes containing aligned sequences of concatenated orthologs were obtained. Based on the obtained core genome sequences of isolates belonging to six STs, a phylogenetic network characterizing the relationships between different NG-MAST types was constructed using the SplitsTree 4.17.1 program (Huson and Bryant, 2006).

### Identification of Genes and Allelic Variants Specific to Each ST

Upon processing the gff files generated with Prokka 1.14.6 using Roary software, a distribution table of genes in the genomes of all 150 analyzed samples (25 samples x 6 STs), consisting of 2856 genes, was compiled. To clarify the function of the identified genes and to divide them into genes and allelic variants, we carried out annotation using pubMLST. To identify genes specific to the isolates of each analyzed ST, the frequencies of the genes in each of the six STs were calculated. First, genes that were identical across all STs, i.e., genes present in at least 80% of the genomes of all STs were removed. Furthermore, by conducting a pairwise comparison of the gene lists for two compared STs, we determined the number of genes present in 80% (in 20 out of 25 genomes) or more of the genomes of isolates belonging to one ST but were absent from or found in no more than 28% (in 7 out of 25 genomes) of the genomes of isolates belonging to another ST. This selection process yielded a list of 329 genes, which are shown in **Table S2** of the **Supplementary Material**. All specific genes identified by Prokka 1.14.6 were annotated with the MPI Bioinformatics Toolkit (https://toolkit.tuebingen.mpg.de) (ProtBLAST, HHpred) (Gabler et al., 2020) to refine gene functions.

### Structural Analysis of GGIs

A sample comprising 150 isolates, i.e., sequences of 125 isolates belonging to STs 225, 1407, 2400, 2992 and 4186, was obtained from the Pathogenwatch database, and the sequences of the 25 Russian isolates of genogroup 807, that we obtained in this work, were analyzed using PubMLST (https://pubmlst.org, section “Single sequence query”) according to the selected typing scheme (“Gonococcal genetic island”). If there was no complete coincidence of the analyzed sequence with the gene sequence in the PubMLST database, for example, if a sequence contained a mutation or stop codon, or the allele was not contained in the database, this sequence was analyzed separately with the choice of a specific locus, for example, locus “NEIS2273(traG)”, “NEIS2275(ych)”, etc., and further checked in the BioEdit program (Ibis Biosciences, Carlsbad, CA) for the presence of mutations, stop codons, or frameshifts.

### Comparison of Drug Resistance of NG-MAST 225, 807, 1407, 2400, 2992, and 4186 Isolates

The data on resistance to azithromycin, cefixime, ceftriaxone, ciprofloxacin, penicillin, and tetracycline for *N. gonorrhoeae* isolates belonging to ST 225 (81 isolates), 1407 (570 isolates), 2400 (269 isolates), 2992 (674 isolates), and 4186 (333 isolates) were obtained from the Pathogenwatch database. For comparison, we used the characteristics of 137 previously studied Russian isolates (Kubanov et al., 2019; Shaskolskiy et al., 2019; Kandinov et al., 2020) belonging to genogroup 807. Isolates with comparisons of their antibiotic susceptibility and their characteristics are listed in **Table S3** of the **Supplementary Material**.

## Results

### Phylogenetic Network of the Relationship Between Isolates of the Studied NG-MAST Types

**Figure 1:**
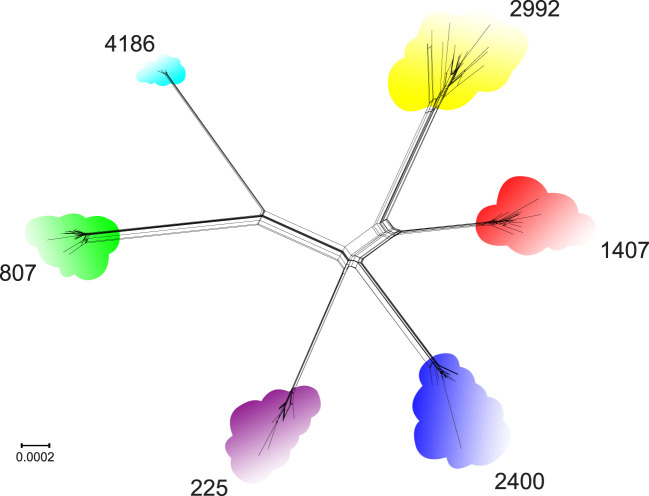
Core genome phylogenetic network of the relationships between *N. gonorrhoeae* genomes of isolates belonging to STs 225, 807, 1407, 2400, 2992 and 4186.

To evaluate the genetic diversity of the six NG-MAST types, a phylogenetic network was constructed based on the genomic data of the corresponding isolates (**Figure 1**). As seen in the figure, the clustering of isolates according to NG-MAST type corresponded to clustering according to the core genomes of *N. gonorrhoeae* STs. The genetic diversity within each ST differed. The greatest diversity was observed for STs 2992 and 2400; the lowest, for ST 4186.

### Structural Comparison of Genetic Gonococcal Islands in Isolates of STs 225, 807, 1407, 2400, 2992, and 4186

**Figure 2:**
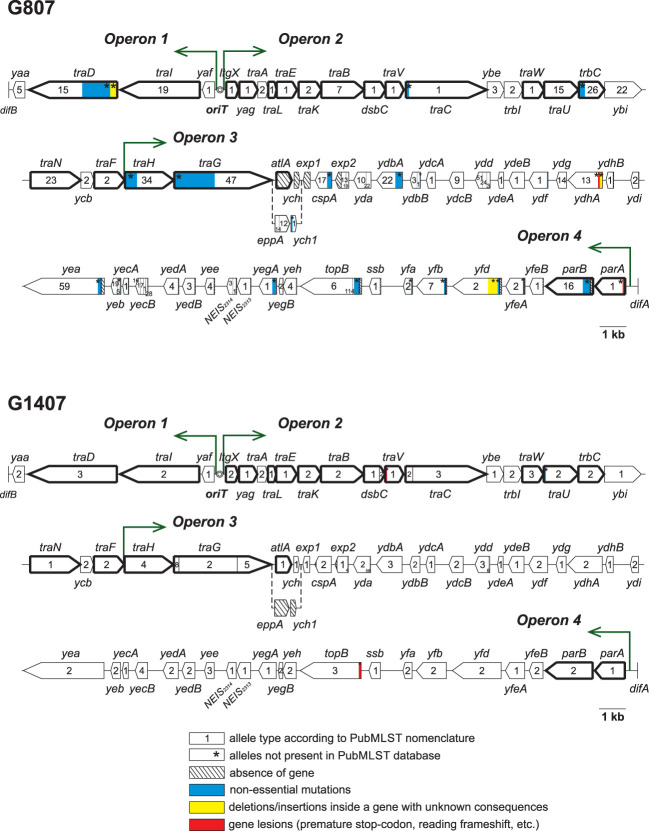
GGI structure of ST 807 and ST 1407 isolates, with definition of genes, alleles and all identified mutations. GGI genes essential for DNA secretion are highlighted in bold.

Detailed sequence analysis of the genes that compose the GGI in isolates of the six STs (25 isolates per ST) revealed significant differences in the structures of gonococcal islands. All identified sequence alterations are described in **Table 2**, and the structures of the GGIs in STs 807 and 1407 with the indicated mutations are shown in **Figure 2**. Both in **Table 2** and in **Figure 2**, GGI genes essential for DNA secretion (Harrison et al., 2016) are highlighted in bold. Allele numbers are given according to the PubMLST nomenclature. Notably, alleles of genes with identified mutations in the GGI genes were not present in the current version of the PubMLST database.

**Table 2:** Differences in the GGI structure in *N. gonorrhoeae* isolates of the six NG-MAST types.

NG-MAST	Presence of GGI	Genes and alleles of GGI according to Pubmlst nomenclature with identified changes in the sequence. Genes essential for DNA secretion are highlighted in bold
225	24 samples of 25	NEIS2292(yea): 2 allele but 1861C→T (stop-codon) (in 5 samples)
807	25 samples of 25	**NEIS2250(traD):** 15 allele but 1256-1257 del(TTTG) (frameshift) (in two samples), 337 G→A (in two samples), 935C→T (in 5 samples), 1399 G→A (in one sample); **NEIS2262(traC):** 1 allele but 1842C →A (in one sample); **NEIS2267(trbC):** 26 allele but 321T→C (in 5 samples); **NEIS2272(traH):** 34 allele but 1403A→C (in one sample) and 727 G→A (in 5 samples); **NEIS2273(traG):** 47 allele but 1195C→T (in 4 samples), 49 allele but 861T→C & 865G →T (in 7 samples); **NEIS2274(atlA): replaced by NEIS2311 (eppA) in all samples;** NEIS2275(ych): replaced by NEIS2312 (ych1) in all samples; NEIS2276(exp1): not found in all samples; NEIS2277(cspA): 83 allele but 252T→C & 274C→T & 476C→T & 583A→T & 584T→A & 586T→A (in 7 samples); NEIS2278(exp2): not found in 7 samples; NEIS2280(ydbA): 3 allele but 571G →A (in 7 samples); NEIS2281(ydbB): 3 allele but 168A→G (in one sample); NEIS2287(ydf): 37 allele but 277A→G (in one sample); NEIS2289(ydhA): 31 allele but 549-560 del(GGCGGATACCTA) (in one sample) and 1064-1074 del (GCGCAATTGAT) (frameshift) (in one sample); excision of all genes from NEIS2289(ydhA) to NEIS2310(parA) (in one sample); NEIS2292(yea): 59 allele but 1839G→A (in one sample); NEIS2293(yeb): 5 allele but 277C→T (in one sample); NEIS2299(yegA): 1 allele but 173A→G (in 5 samples); NEIS2302(topB): 114 allele but 278C→T (in two samples); NEIS2304(yfa): 2 allele but 251 delT (in one sample); NEIS2305(yfb): 7 allele but 11C→T (in one sample); NEIS2306(yfd): 2 allele but 385G→T (in one sample), 263A→G & 855-877 del(TGATTGGCGGAAAGGGCCAAATG) (in 5 samples); NEIS2307(yfeA): 2 allele but 114A→G (in one sample); **NEIS2309(parB):** 16 allele but 363C→T (in 5 samples); NEIS2312(ych1): 8 allele but 151C→T & 155G→A & 159C→A & 162T→ C (in 7 samples).
1407	24 samples of 25	**NEIS2261(traV):** 30% of gene truncated (in one sample); **NEIS2266(traU):** 2 allele but 703A→G (in one sample); NEIS2277(cspA): not found (in one sample); NEIS2278(exp2): not found (in two samples); NEIS2302(topB): 15% of gene truncated (in one sample).
2400	25 samples of 25	No mutations found
2992	4 samples of 25	**NEIS2271(traF):** 2 allele but 54T→G (in one sample); **NEIS2274(atlA): replaced by NEIS2311 (eppA)** (in one sample); NEIS2275(ych): replaced by NEIS2312 (ych1) (in one sample); NEIS2276(exp1): not found (in one sample); NEIS2278(exp2): 5 allele but 338-339 del(TTCA) & 518 G→A (frameshift) (in one sample); NEIS2284(ydd): 6 allele but 368C→T (in one sample); NEIS2292(yea): 8 allele but 300T→C (in one sample).
4186	24 samples of 25	**NEIS2251(traI): 2 allele but 1153G→T (stop-codon) in all samples;** **NEIS2258(traK):** 1 allele with 300 delT (frameshift) (in one sample); **NEIS2261(traV):** 89% of gene truncated (in one sample); **NEIS2265(traW):** not found (in one sample); **NEIS2269(traN):** not found (in one sample); NEIS2277(cspA) and NEIS2278(exp2): not found in all samples; NEIS2281(ydbB): 17 allele but 221G→A (in 3 samples); NEIS2284(ydd): 40 allele (this allele has already 6 stop-codons) but 430G→A in all samples); NEIS2293(yeb): not found (in one sample); NEIS2294(yecA): not found (in one sample); NEIS2295(yecB): 23% of gene truncated (in 4 samples); NEIS2296(yedA): 2 allele but 342 insC (frameshift) in all samples; NEIS2299(yegA): 1 allele but 274 delA (frameshift) (in 3 samples); NEIS2302(topB): 56 allele but 1946G→A (in one sample); NEIS2306(yfd): 2 allele but 1280T→G (in one sample).

Genes essential for DNA secretion are highlighted in bold.

Not all studied isolates contained a GGI. Most isolates belonging to STs 225, 807, 1407, 2400, and 4186 (24-25 of 25) contained a GGI; however, in isolates of ST 2992, a GGI was present in only 4 of 25 samples.

In the isolates of ST 225, all GGI genes were intact (non-mutated), indicating that they were functioning properly. An exception was the putative helicase gene *yea*, in which the 1861C→T substitution was found in five samples, leading to the formation of a premature stop codon (**Table 2**). However, the *yea* gene is nonessential for GGI function (Harrison et al., 2016).

In the ST 807 isolates, significant changes were revealed in genes both essential and nonessential for GGI function. The identified key change was the replacement of the essential *atlA* gene with the *eppA* gene, which occurred in all 25 isolates and was accompanied by the replacement of the *ych* gene with the *ych1* gene and the absence of the *exp1* gene. In some isolates of ST 807, changes in *traG* alleles also occurred, i.e., mutation of NEIS2273(traG) allele 47 or replacement of allele 47 with allele 49, accompanied by mutation (**Table 2**). These changes can result in the loss of the T4SS DNA secretion ability, as, for example, was shown by Kohler et al. (2013) and Pachulec et al. (2014). Interestingly, although the function of the proteins encoded by the *atlA* (autolysin A, peptidoglycan transglycosylase), and *eppA* (endopeptidase, M23 metallopeptidase) genes is cleavage of the peptidoglycan during the formation of a pore in the bacterial cell wall for installing the T4SS, the Epp protein cannot replace AtlA, for an unknown reason (Kohler et al., 2013).

The isolates of ST 1407 showed a small number of changes in both essential and nonessential GGI genes, and all the changes were observed in only one or two of the 25 samples. In the ST 2400 samples, all GGI genes were intact and appeared to be functioning.

Only a small fraction of the ST 2992 samples carried the GGI. One sample with the GGI harbored substitution of the *atlA* gene with the *eppA* gene, which could lead to loss of the functional activity of the GGI.

The isolates of ST 4186 carried several mutations in essential and nonessential GGI genes. The most important change was the presence of a stop codon in the important *traI* gene, encoding the relaxase, in all 24 GGI-harboring samples; thus, none of these isolates were likely to be able to secrete ssDNA.

Thus, although 126 (84%) of the studied isolates in the sample carried a GGI in their genome, only 75 of them (59.5%) were potentially capable of secreting ssDNA.

### Antimicrobial Resistance of *N. gonorrhoeae* Isolates of Different STs and Its Association With GGI Structure

Next, we investigated the susceptibility of isolates of different STs to antimicrobial drugs, including antimicrobials currently used (azithromycin, ceftriaxone, and cefixime) and those previously used (ciprofloxacin, penicillin, and tetracycline) to treat gonococcal infections. Data on the drug susceptibility of ST 225, 1407, 2400, 2992, and 4186 isolates obtained from the Pathogenwatch database and data on the Russian isolates of ST 807 were used, for 2064 isolates in total. The ratio of drug-resistant isolates differed across the STs (**Table 3**; initial data are provided in **Table S3** of the **Supplementary Material**).

**Table 3:** Antimicrobial susceptibility of *N. gonorrhoeae* isolates belonging to different STs.

ST	Intermediately resistant and Resistant* (% of total amount of analyzed isolates)					
	Azithromycin	Ceftriaxone	Cefixime	Ciprofloxacin	Penicillin	Tetracycline
ST 225	0 and 1.2	0 and 0	0 and 0	0 and 100	87.7 and 12.3	1.2 and 98.8
ST 1407	0 and 2.1	0 and 0.7	0 and 94.0	0 and 99.6	1.2 and 98.8	0.9 and 99.1
ST 2400	0 and 1.1	0 and 0	0 and 5.2	0 and 99.6	91.1 and 8.9	0 and 100
ST 2992	0 and 8.8	0 and 0	0 and 0.3	0.1 and 0.4	96.4 and 3.6	96.6 and 3.4
ST 4186	0 and 0.3	0 and 0	0 and 1.2	0 and 0	99.1 and 0.6	0.9 and 0
ST 807	0 and 0.7	0 and 0	–	0 and 2.2	46.7 and 0.7	2.9 and 0.7

*According to the European Committee on Antimicrobial Susceptibility Testing (EUCAST).

Most ST 807 and ST 4186 isolates were susceptible to antimicrobials except for penicillin. No data on the resistance of ST 807 isolates to cefixime were available since this drug is almost never used for the treatment of gonococcal infection in the Russian Federation. The most resistant isolates were those of ST 1407, almost all of which were resistant to cefixime, ciprofloxacin, penicillin, and tetracycline. Isolates of this ST, which is widespread worldwide, carry multiple drug resistance determinants, in particular, nucleotide substitutions in the *porB* gene encoding the porin protein that impede the entry of antibiotics into the cell and a deletion in the promoter region of the pump regulator gene *mtrR* that leads to increased efflux of antibiotics; moreover, most isolates of ST 1407 carry a mosaic allele of the penicillin-binding protein gene *penA* (Unemo and Shafer, 2014; Unemo and Jensen, 2017; Unemo et al., 2019).

Thus, phylogenetically different STs (**Figure 1**) also differ in antibiotic resistance profiles (**Table 3**), which can be explained not only by the specifics of antimicrobial drug use in the countries where the isolates were collected but also by differences in evolutionary and adaptation pathways of the considered STs that allow them to survive and occupy a prominent position in the gonococcal population.

**Figure 3:**
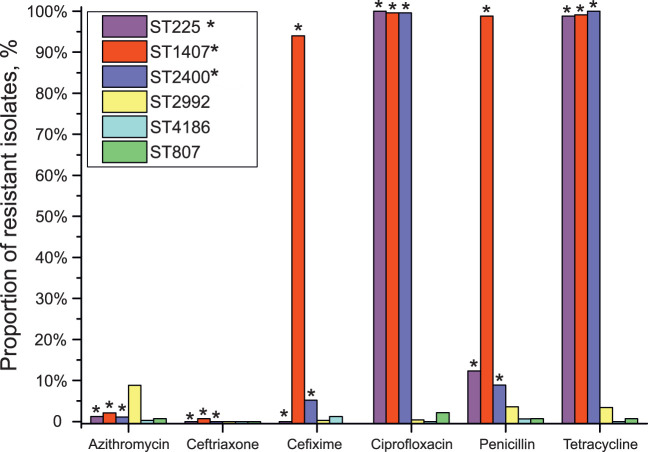
Association between GGI function and the resistance to azithromycin, ceftriaxone, cefixime, ciprofloxacin, penicillin, and tetracycline for isolates belonging to STs 225, 807, 1407, 2400, 2992 and 4186. No data on the resistance of ST 807 isolates to cefixime are available. STs carrying a GGI without lesions in genes essential for GGI function (“properly functioning GGI”) are marked with asterisks.

Assuming that the GGI structural regularities found for the studied STs were preserved, we compared the drug susceptibility of isolates of different STs with the presence of a “properly functioning” GGI, i.e., a GGI without lesions in the essential genes (**Figure 3**). As seen in the figure, isolates of STs 225, 1407, and 2400 with a “properly functioning” island without obvious lesions/critical changes in essential genes (premature stop codons or reading frameshifts) demonstrated increased resistance to ciprofloxacin, penicillin, and tetracycline. Isolates of STs 1407 and 2400 were also resistant to cefixime. On the other hand, most isolates carrying gene lesions or not containing a GGI, e.g., isolates of STs 807, 2992 and 4186, did not show an increase in resistance to these antibiotics. However, it should be mentioned that the proportion of isolates resistant to azithromycin was higher for the isolates without “properly functioning” GGI (8.8% azithromycin-resistant isolates for ST 2992).

Grouping of STs according to the presence/absence of a “properly functioning” GGI (a group containing STs 225, 1407 and 2400 and a group containing STs 807, 2992 and 4186) and analysis with the χ-square test revealed differences (p <0.001) in susceptibility to cefixime, ciprofloxacin, penicillin and tetracycline between isolates carrying a GGI without lesions in essential genes and isolates either without a GGI or carrying a GGI with lesions in essential genes. Importantly, we considered the association of resistance not with the presence of a GGI itself but with the presence of a “properly functioning” GGI without lesions in genes essential for its function.

### Genomic Comparison of Isolates Belonging to Different STs and Identification of Genes Specific to Each ST

Analysis and annotation of genes in the genomes of 150 N*. gonorrhoeae* isolates belonging to STs 225, 807, 1407, 2400, 2992, and 4186 using the Prokka program showed the presence of 2856 genes in the pangenome. Annotation with pubMLST allowed us to separate genes and allelic variants and to clarify gene functions. After analysis with the pubMLST program, 2149 genes remained in the pangenome. Among these 2149 genes, 1799 were present in at least 80% of the genomes, 164 were present in more than 15% but less than 80% of the genomes, and 184 were present in less than 15% of the genomes.

**Figure 4:**
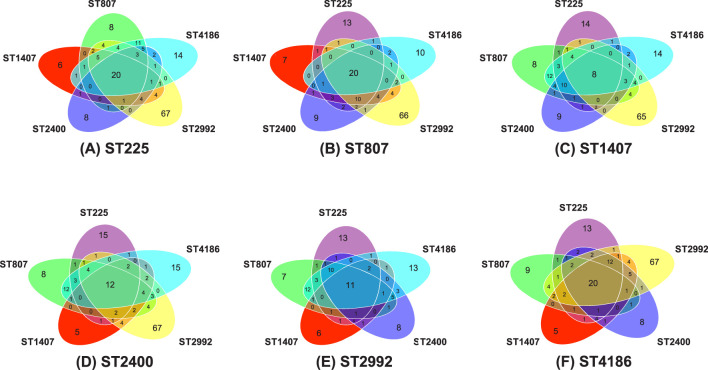
Venn diagrams characterizing pairwise comparisons of the genomes of isolates belonging to one ST with the genomes of isolates belonging to the five other STs. Reference STs: ST 225 **(A)**, ST 807 **(B)**, ST 1407 **(C)**, ST 2400 **(D)**, ST 2992 **(E)** and ST 4186 **(F)**. The numbers on the diagrams indicate the numbers of genes present in at least 80% of the reference ST isolates and not detected or present in no more than 28% of isolates belonging to another ST. The center of each diagram shows the number of genes that are specific to the indicated ST but are minor components in the five other STs.

To identify genes specific to a given ST, we performed a pairwise comparison of ST genomes using the following parameters: the number of genes (determined using the Prokka program) present in at least 80% of the genomes of isolates belonging to one ST and either were not detected at all or were detected in no more than 28% of the genomes of isolates belonging to another ST (minor components). The combined pairwise comparisons of the genomes of the six STs are shown in **Figure 4** as Venn diagrams. For example, comparison of the genomes of ST 807 isolates with the genomes of other ST isolates (**Figure 4B**) showed the following:

- 13 genes were absent or were minor components in isolates of ST 225 but were present in isolates of the five other STs;
- 10 genes were absent or were minor components in isolates of ST 4186 but were present in isolates of the five other STs;
- 66 genes were absent or were minor components in isolates of ST 2992 but were present in isolates of the five other STs;
- 9 genes were absent or were minor components in isolates of ST 2400 but were present in isolates of the five other STs;
- 7 genes were absent or were minor components in isolates of ST 1407 but were present in isolates of the five other STs;
- 20 genes were specific to ST 807 but were minor components in isolates of the five other STs.

As a result of 30 pairwise comparisons of the genomes of the six STs, 329 genes specific to one ST but minor components in another ST were identified. All 329 genes are listed in **Table S2** of the **Supplementary Material**. Intersections of the lists of pairwise comparisons revealed genes specific to a particular sequence type, 8-20 genes for each sequence type (**Table 4**). Thus, **Table 4** presents a list of genes specific to one ST but not specific to any of the other five STs and the functions of the proteins encoded by these genes that were revealed by annotation. The list of genes includes the specific alleles of the *tbpB* gene encoding transferrin binding protein B (TbpB), which determine an NG-MAST type and have been identified for all STs.

**Table 4:** Loci and gene alleles specific to the analyzed STs and the functions of the proteins encoded by these genes.

PubMLST locus: allele id numbers	PubMLST product (match)	Prokka annotation
**ST 225:**		
NEIS0210 (pilE): 596	fimbrial protein pilE*	pilin
NEIS0210 (pilE): 787	fimbrial protein pilE*	pilin
NEIS0829 (pilJ): 593	type IV biogenesis protein PilJ**	PilW family protein
NEIS0828 (pilI): 386	type IV biogenesis protein PilI**	type IV pilus modification protein PilV
NEIS0827 (pilH/fimT): 1233	type IV biogenesis protein (PilH/FimT)**	Tfp pilus assembly protein FimT/FimU
NEIS1691(tbpb): 723 (NG-MAST_tbpb: 4)	tbpB (NG-MAST)*	tbpB (NG-MAST)
NEIS2016: 937	hypothetical protein*	M48 family metallopeptidase
NEIS1058: 29	phage tail fiber protein* (no start codon)	hypothetical protein
NEIS2937: 8	hypothetical protein*	tRNA nuclease CdiA-2
NEIS0586: 980	MafB toxin, МafB1 MGI-2*	hypothetical protein
NEIS2361: 6	NgoAV type I restriction-modification system; HsdM; DNA methyltransferase subunit M* (internal stop codon)	type I restriction-modification system, subunit M
NEIS3188: 1	type IV toxin-antitoxin system, putative AbiEii toxin	ATP-binding protein
NEIS1796: 607	hypothetical protein**	immunity 41 family protein
NEIS0908: 349	transmembrane transport protein*	хanthine permease XanQ
NEIS2603: 49	putative phage-associated protein*	hypothetical protein
NEIS1661: 99	phage replication initiation protein*	hypothetical protein
NEIS1265: 154	putative protein (PubMLST, Prokka); sel1 repeat family protein (ProtBLAST, HHPred)	
NEIS1265: 394	hypothetical protein (PubMLST, Prokka); sel1 repeat family protein (ProtBLAST, HHPred)	
NEIS1265: 449	putative protein (PubMLST, Prokka); sel1 repeat family protein (ProtBLAST, HHPred)	
NEIS1254: 13	hypothetical protein	
**ST 807:**		
NEIS0831 (pilX): 437	minor pilin PilX**	hypothetical protein
NEIS0827 (pilH/fimT): 1230/1245	type IV biogenesis protein, РilH/FimT**	Tfp pilus assembly protein FimT/FimU
NEIS0828 (pilI): 120	type IV biogenesis protein, PilI**	type IV pilus modification protein PilV
NEIS0829 (pilJ): 4770/1074/24/1508	type IV biogenesis protein pilJ **	PilW family protein
NG-MAST_tbpb: 27	tbpB (NG-MAST)**	tbpB (NG-MAST)
NEIS2083: 190 (NEISp2083: 98)	MafA3 lipoprotein, МafA MGI-3**	adhesin MafA
NEIS2273(traG):47/49, **GGI component**	TraG, pilus assembly; mating-pair stabilization protein*	conjugal transfer protein TraG
NEIS2311(eppA):12/14, **GGI component**	hypothetical protein eppA**	M23 family metallopeptidase
NEIS2312 (ych1): 1/8, **GGI component**	hypothetical protein ych1	hypothetical protein
NEIS2016: 586	hypothetical protein* (internal stop codon)	M48 family metallopeptidase
NEIS1439: 16	putative nuclease*	thermonuclease family protein
NEIS1310(modA): 954	NgoAXII type III restriction-modification system, methylase ModA*	site-specific DNA-methyltransferase
NEIS0263: 553	hypothetical protein* (internal stop codon)	ComF family protein
NEIS3174: 2 (NEIS3201: 2)	AAA family ATPase virulence protein**	hypothetical protein
NEIS0916: 46	lipoprotein*	hypothetical protein
NEIS0813: 983	perisplasmic protein	hypothetical protein
NEIS0595: 48 (NEIS 0608: 93)	hypothetical protein	hypothetical protein
NEIS2078: 99 (NEIS1241(adhC): 48)	hypothetical protein	hypothetical protein
NEIS0535: 667	hypothetical protein	hypothetical protein
NEIS2979: 132	hypothetical protein	hypothetical protein
**ST 1407:**		
pilS: 26	pilS cassette region*	hypothetical protein
pilS: 33	pilS cassette region*	hypothetical protein
NEIS0210 (pilE): 851	fimbrial protein PilE*	pilin
NEIS1691 (tbpb): 734 (NG-MAST_tbpb:110)	tbpB (NG-MAST)**	tbpB (NG-MAST)
NEIS1719(opaA): 668 (NEIS1403: 660)	opacity protein*	outer membrane beta-barrel protein
NEIS1753 (penA): 266/327/531	penicillin-binding protein 2, PenA**	penicillin-binding protein 2 PenA
NEIS0593: 62	hypothetical protein**	HNH endonuclease, MafB family polymorphic toxin
NEIS1876: 21	hypothetical protein	hypothetical protein
**ST 2400:**		
pilS: 26	pilS cassette region*	hypothetical protein
pilS: 33	pilS cassette region*	hypothetical protein
NEIS0210 (pilE):1834	fimbrial protein PilE*	pilin
NEIS0210 (pilE):2493	fimbrial protein PilE*	pilin
NEIS0210 (pilE): 339/789/1355	fimbrial protein PilE*	pilin
NEIS0210 (pilE):1906	fimbrial protein PilE*	pilin
NEIS1691(tbpb):1877 (NG-MAST_tbpb: 563)	tbpB (NG-MAST)**	tbpB (NG-MAST)
NEIS1929: 47	acetyltransferase**	hypothetical protein
NEIS1706: 744	integral membrane efflux protein*	multidrug efflux MFS transporter
NEIS0261: 326	putative periplasmic protein**	septal ring lytic transglycosylase RlpA family protein
NEIS1366: 993 (NEIS0932(sucB): 63)	3-phosphoshikimate 1-carboxyvinyltransferase*	HAD hydrolase family protein
NEIS2723: 2	hypothetical protein	hypothetical protein
**ST 2992:**		
NEIS0827 (pilH/fimT): 573	type IV biogenesis protein PilH/FimT*	Tfp pilus assembly protein FimT/FimU
NG-MAST_tbpb: 29	tbpB (NG-MAST)**	tbpB (NG-MAST)
NEIS3211: 1	helix-turn-helix transcriptional regulator**	hypothetical protein
NEIS0331: 5	DNA-binding protein**	DNA-binding transcriptional regulator
NEIS1290 (gatC): 102/187	aspartyl/glutamyl-tRNA(Asp/Gln) amidotransferase, subunit C*	Asp-tRNA(Asn)/Glu-tRNA(Gln) amidotransferase subunit GatC
NEIS1633 (mtrD): 1006	drug efflux protein**	multidrug efflux RND transporter permease subunit MtrD
NEIS1400: 98	putative integral membrane transporter*	hypothetical protein
NEIS0594: 9	hypothetical protein	hypothetical protein
NEIS0332: 8	hypothetical protein	hypothetical protein
NEIS0938: 87	hypothetical protein	hypothetical protein
NEIS1958:252	hypothetical protein	hypothetical protein
**ST 4186:**		
NEIS0829 (pilJ):1989	type IV biogenesis protein**	PilW family protein
NEIS0827 (pilH/fimT): 1232	type IV biogenesis protein PilH/FimT**	Tfp pilus assembly protein FimT/FimU
NEIS0371 (pilC1):272	type IV pilus associated protein PilC1*	hypothetical protein
NEIS0371 (pilC1): 94	type IV pilus associated protein PilC1*	hypothetical protein
NEIS2273 (traG):142, **GGI component**	TraG, pilus assembly; mating-pair stabilization protein*	hypothetical protein
NEIS1691(tbpb):1953 (NG-MAST_tbpb:241)	tbpB (NG-MAST)**	tbpB (NG-MAST)
NEIS1777 (mapA): 53	maltose phosphorylase MapA*	maltose phosphorylase
NEIS1085: 999 (NEIS0502: 149)	heat shock protein GrpE*	hypothetical protein
NEIS1007: 8	hypothetical protein*	Smr/MutS family protein
NEIS1669: 80	hypothetical protein*	roadblock/LC7 domain-containing protein
NEIS2016: 589	hypothetical protein**	M48 family metallopeptidase
NEIS0593: 99	hypothetical protein**	HNH endonuclease
igr_upNEIS1364: 97	intergenic region*	hypothetical protein
NEIS2615: 72/59	putative phage associated protein	hypothetical protein
NEIS2685: 5	putative phage associated protein	hypothetical protein
NEIS2710: 241	putative phage associated protein	hypothetical protein
NEIS3192: 6/7	hypothetical protein	hypothetical protein
NEIS0728: 5	hypothetical protein	hypothetical protein
NEIS3210: 1	hypothetical protein	hypothetical protein
NEIS3199: 9	hypothetical protein	hypothetical protein

*Closest match, **exact match. GGI components are highlighted in bold.

For all studied STs, variable alleles of genes encoding pili proteins (PilE, PilH, PilX, PilJ, etc.) were identified as specific alleles; these genes are highly variable in *N. gonorrhoeae*. As seen in **Table 4**, the genes specific to each ST also include genes encoding hypothetical proteins and proteins whose functions have not been established.

For ST 225, the specific genetic loci included genes encoding the transmembrane transport protein xanthine/uracil permease XanQ; the putative microbial toxin AbiEii; which induces cell death during phage infection; type IV toxin-antitoxin system (Dy et al., 2014); a MafB family polymorphic toxin involved in interbacterial competition (Jamet and Nassif, 2015; Arenas et al., 2020); the type I restriction-modification system protein NgoAV; and a protein of the sel1 repeat family associated with upregulation of a set of genes negatively regulated by ferric uptake regulator (Fur) and downregulation of a set of genes positively regulated by Fur (Li et al., 2017).

The genes specific to ST 807 included a gene encoding the multiple adhesin family protein MafA, whose expression products are involved in virulence, adhesion and transcytosis in pathogenic *Neisseria* and are required for the function of the polymorphic toxin MafB (Arenas et al., 2020); a gene encoding a protein of the ComF family and required for transformation (Tribble et al., 2012); an M48 family metallopeptidase gene; a gene encoding a protein of the thermonuclease family belonging to the replication, recombination and repair system; a gene encoding the modification methylase ModA (restriction-modification system); which is involved in the control of phase variations in representatives of the *Neisseria* genus (Gawthorne et al., 2012); and a gene encoding an AAA family ATPase that may act as a toxin for a type IV toxin-antitoxin resistance system (Yamamoto et al., 2009). We also identified a number of specific genes that constituted the GGI, in particular, *traG*, *eppA*, and *ych1*, in which alterations or lesions have occurred (premature stop codons or reading frameshifts).

For ST 1407, the specific genes included a type XXXIV mosaic allele of the *penA* gene encoding the PBP 2 protein, whose presence reduces the susceptibility of *N. gonorrhoeae* to β-lactam drugs, and genes encoding the opacity protein and the MafB family polymorphic toxin HNH endonuclease (Jamet and Nassif, 2015; Arenas et al., 2020).

The list of genes specific to ST 2400 included a gene for an acetyltransferase involved in lipid A biosynthesis (Bartling and Raetz, 2009); a gene encoding an efflux pump protein of the major facilitator superfamily (MFS) that carry out transfer of small molecules across cell membranes (Bosshart and Fotiadis, 2019); a gene encoding the lytic transglycosylase RlpA, which exhibits unusual specificity for naked glycans and is necessary for cell division and the correct biogenesis of the bacterial cell wall (Jorgenson et al., 2014); and a gene encoding the phosphoshikimate 1-carboxyvinyltransferase enzyme of the HAD hydrolase family.

The following specific genes were identified for ST 2992: a gene encoding the phage transcriptional regulator of the “helix-turn-helix” motif, a gene encoding the DNA-binding transcriptional regulator protein, a gene encoding subunit C of aspartyl/glutamyl-tRNA amidotransferase, and a gene encoding the permease subunit of the RND-type MtrD efflux pump associated with the resistance to penicillins, tetracyclines, macrolides (azithtomycin) and cephalosporins (Shafer et al., 2016).

For ST 4186, the specific genes included the gene encoding the maltose phosphorylase MapA, which is involved in maltose metabolism; a gene encoding an Smr/MutS family protein involved in DNA repair (Fukui and Kuramitsu, 2011); a gene encoding the heat shock protein GrpE; a gene encoding an M48 family metallopeptidase; and the specific allele 142 of the *traG* gene, a GGI component.

### Genomic Comparison of ST 807 and ST 1407

The pairwise comparison of the genomes of ST 807, which is the most widespread ST in the Russian Federation, and the genomes of ST 1407, which is otherwise the most common ST worldwide but not in the Russian Federation, was particularly interesting. This comparison revealed 53 loci for ST 807 and 62 specific loci for ST 1407, including pseudogenes and genes encoding hypothetical proteins. First, notably, the ST 807 isolates carried a type I nonmosaic allele of the *penA* gene, while the ST 1407 isolates carried a type XXXIV mosaic allele associated with resistance to third-generation cephalosporins.

As shown above, the structure of the GGI differed between ST 807 and ST 1407 (**Figure 2**). In both STs, an allele of the *traG* gene specific to that type was found. However, unlike the ST 807 genome, the ST 1407 genome contained the *atlA* gene instead of the *eppA* gene, which may contribute to the pathogenesis of gonococcal infection (Kohler et al., 2013; Pachulec et al., 2014).

The genomes of both STs were characterized by specific genes and alleles encoding proteins that are components of pili and proteins involved in pilus biogenesis (*pilS*, *pilE*, *pilV*, *pilI*, *pilJ*, *pilX*, *pilH*, *pilV*); unique alleles of genes encoding pathogenicity factors, including the opacity protein OpaA (which interacts with receptors of the CEACAM family); and specific alleles of genes encoding proteins of the ComF family that are important for the uptake of exogenous DNA by naturally competent bacteria. The genomes of both STs contained specific proteins responsible for the initiation of replication; a characteristic ATP-dependent chaperone, ClpB; a 7-carboxy-7-deazaguanine synthase, QueE; a metallopeptidase of the M48 family; and a protein of the thermonuclease family.

Proteins that were present in most ST 1407 samples but were absent from or minor components in the ST 807 samples included an HNH endonuclease of the MafB family, an Eco29kI family restrictase, a DNA (cytosine-5)-methyltransferase and type II toxin-antitoxin system VapC family toxin, and proteins of the transport and efflux systems, including a HlyD family efflux transporter protein and a peptidase domain-containing ABC transporter.

Proteins that were present in most ST 807 samples but were absent from or minor components in the ST 1407 samples included MafA adhesins; immunity 8 family protein, a toxin belonging to the most common family of immunity proteins whose C-terminal domain is characterized by significant variability due to recombination with genes encoding other toxins (Zhang et al., 2012); enzymes in biochemical pathways, including glycosyl transferases (protein glycosylation system); a Zn-dependent alcohol dehydrogenase; and a restriction endonuclease of the NgoFVII family.

## Discussion

In this work, the complete genomes of 25 Russian isolates belonging to ST 807 and closely related STs were obtained for the first time. Genogroup G807 has been predominant in the Russian Federation over the past 10 years, and its notable feature is its susceptibility to antimicrobial drugs (Kubanov et al., 2019; Kandinov et al., 2020; Shaskolskiy et al., 2020a). The genomes of *N. gonorrhoeae* isolates belonging to the NG-MAST types that are the most widespread worldwide (ST 225, 1407, 2400, 2992, and 4186) and in the Russian Federation (ST 807) were compared. The constructed phylogenetic network for 25 core genomes of each ST showed that the clustering of the isolates according to NG-MAST type corresponded to the clustering according to the core genome, consistent with the results of Harrison et al. (2020). Thus, the NG-MAST scheme provides information about and correctly describes the phylogeny of *N. gonorrhoeae* isolates as revealed by WGS. Database analysis showed that phylogenetically different STs also differed in the profiles of resistance to the drugs that are currently and were previously used for gonorrhea treatment.

The genomes of *N. gonorrhoeae* isolates of different STs were compared to identify genes that distinguish one sequence type from the others. To compare genomes, the following criteria were chosen: genes and gene alleles present in at least 80% (that is, in 20 out of 25 genomes under study) of the genomes of isolates belonging to one ST and either were absent from or were present in no more than 28% (that is, in 7 out of 25 genomes under study) of the genomes of isolates belonging to another ST. For comparison, in the work of Golparian et al. (2020), when the entire Danish gonococcal population for the period from 1928 to 2013 was analyzed, the corresponding criteria of ≥ 99% and less than 15% were applied. In our work, we analyzed a much smaller population containing only individual STs; moreover, we sought to avoid information loss due to errors in the sequencing results.

The differences in the genomes of *N. gonorrhoeae* isolates belonging to different NG-MAST types included differences in a large set of genes and gene alleles: the genetic lines of the studied STs contained from 8 to 20 different genes specific to each of these types. The functions of specific genes differed, with the exception of the genes encoding pili proteins, which were found in all STs; this similarity is predictable given the pathogenic role of pili and their phase variations. Analysis of the set of genes that distinguished individual NG-MAST types showed the presence of genes associated with host adaptation. In particular, ST 225 was characterized by the presence of MafB, a toxin for the defense against other bacteria, as well as a protein of the Sel1 repeat family, which plays an important role in iron metabolism. For type 807, the *mafA* gene, whose expression products are involved in virulence, adhesion and transcytosis in pathogenic *Neisseria* and are necessary for the function of MafB (Arenas et al., 2020), was notable.

Analysis of genes specific for each ST indicated the presence of two genes contributing to the antibiotic resistance of *N. gonorrhoeae*: a mosaic allele of *penA* gene in ST 1407, that is strongly associated with resistance to cephalosporins, and an *mtrD* gene of multidrug efflux transporter in ST 2992 associated with the resistance to penicillins, tetracyclines, macrolides (azithtomycin) and cephalosporins. The presence of specific *mtrD* gene in isolates of ST 2992 may contribute to the increased resistance to azithromycin (**Figure 3** and **Table 3**).

Harrison et al., on the basis of WGS data for 3750 N*. gonorrhoeae* isolates, obtained core genomes and identified the most conserved and differing genomic loci (Harrison et al., 2020). The most conserved loci were NEIS0415 (ribosome biogenesis GTP-binding protein YsxC) and NEIS2686 (hypothetical protein). The less conserved loci included those associated with pilin biosynthesis (NEIS0827), cell division (NEIS0116 and NEIS0128), iron acquisition (NEIS0338), and MafA proteins in the toxin-antitoxin system. Several genes that we identified as specific coincided with the genes described earlier (Harrison et al., 2020), for example, the gene encoding a type IV biogenesis protein (NEIS0827) in ST 225 and the *mafA* gene in ST 807 (see **Table 4**).

A detailed study of the GGI gene sequences showed significant differences in the GGI structure between isolates belonging to different STs. Isolates of ST 807 and ST 4186 harbored mutations in the GGI genes, which play an essential role in DNA secretion, leading to loss of functionality. In all isolates of ST 807, the *atlA* gene, which is critically important for GGI function, was replaced with the *eppA* gene, accompanied alteration of the *traG* allele, replacement of the *ych* gene with the *ych1* gene, and loss of the *exp1* gene. Only a small proportion of ST 2992 isolates (4 of 25) carried GGI genes.

The isolates of STs 225, 1407, and 2400 lacked significant lesions in the essential GGI genes, i.e., these isolates are predicted to secrete DNA. The proportion of isolates resistant to ciprofloxacin, penicillin, and tetracycline among isolates with STs DNA secretion capability was significantly higher than that among isolates of STs without a GGI or with a GGI harboring lesions in essential genes. Thus, our results indicated the presence of a relationship between the decrease in the susceptibility of isolates to ciprofloxacin, penicillin, and tetracycline and the absence of lesions in the GGI genes necessary for DNA secretion.

The association between the presence of a GGI and genotype-predicted resistance to numerous antibiotics, including cefixime, ciprofloxacin, and penicillin, was noted earlier by Harrison et al, 2016. However, as our results indicate, simply the presence of a GGI, which is found in ~80% of *N. gonorrhoeae* isolates, does not guarantee its proper function for ssDNA secretion, and lesions/changes in the key genes that could interfere with the entire T4SS system must be considered.

Thus, WGS indicated genomic differences between isolates belonging to the most common NG-MAST types. The evolutionary pathways of *N. gonorrhoeae*, which allow a particular NG-MAST type to maintain long-term predominance in the population for many years, may include changes in genes responsible for adhesion and virulence, changes in the GGI structure, preservation of genes that carry the determinants of drug resistance, and changes in genes associated with host adaptation and genes encoding enzymes of biochemical pathways. In-depth analysis of the significance of such differences in the ongoing evolution of *N. gonorrhoeae* is undoubtedly required. However, the results obtained in this work for the comparison of this limited number of genomes (25 genomes of each of the six STs) provide information on the presence of genes specific to each ST, which can be used to create *in vivo* and *in vitro* models of the pathogenesis of gonococcal infection.

## Data Availability Statement

The datasets presented in this study can be found in online repositories. The names of the repository/repositories and accession number(s) can be found in the article/**Supplementary Material**.

## Ethics Statement

Ethical approval/written informed consent was not required for the study of animals/human participants in accordance with the local legislation and institutional requirements.

## Author Contributions

BS designed and directed the project and performed bioinformatic studies. DK, IK, SG, and BS carried out WGS and analyzed WGS results. DK analyzed the structures of gonococcal genetic islands. AK supervised *N. gonorrhoeae* sample collection. VS and DD carried out NG-MAST typing and preparation of samples for WGS. ED carried out gene annotation and wrote the manuscript. DG designed and supervised the project and wrote the manuscript. All authors contributed to the article and approved the submitted version.

## Funding

This work was supported by the Russian Science Foundation, grant number 17-75-20039 (WGS and analysis of genomes) and by the Ministry of Science and Higher Education of the Russian Federation to the EIMB Center for Precision Genome Editing and Genetic Technologies for Biomedicine under the Federal Research Program for Genetic Technologies Development for 2019-27, agreement number 075-15-2019-1660 (analysis of drug resistance of *N. gonorrhoeae* isolates). Collection and susceptibility testing of *N. gonorrhoeae* clinical isolates were performed according to the Ministry of Health of the Russian Federation assignment number 056-03-2021-124.

## Conflict of Interest

The authors declare that the research was conducted in the absence of any commercial or financial relationships that could be construed as a potential conflict of interest.

## Publisher’s Note

All claims expressed in this article are solely those of the authors and do not necessarily represent those of their affiliated organizations, or those of the publisher, the editors and the reviewers. Any product that may be evaluated in this article, or claim that may be made by its manufacturer, is not guaranteed or endorsed by the publisher.
