# Supplementary material for: Comparative Whole-Genome Analysis of Neisseria gonorrhoeae Isolates Revealed Changes in the Gonococcal Genetic Island and Specific Genes as a Link to Antimicrobial Resistance
Source: Front Cell Infect Microbiol. 2022 Feb 18;12:831336. doi: 10.3389/fcimb.2022.831336 (PMC8895040; doi:10.3389/fcimb.2022.831336)
Supplement: Supplementary file 1 [file Table_1.docx]

Table S1. List of *N. gonorrhoeae* samples taken from the Pathogenwatch database (https://pathogen.watch/genomes/ all? OrganismId = 485) belonging to NG-MAST types 225, 1407, 2400, 2992, and 4186 used the for comparative whole genome analysis

| № | Name | Sample ID | Year | NG-MAST | MLST | Country |
| --- | --- | --- | --- | --- | --- | --- |
| 1 | ERS135373 | 58ac238772251e0001022a30 | 2010 | 225 | 1901 | USA |
| 2 | ERR234474 | 5e4bb0cac09ca0b7a5f8eb85 | 2004 | 225 | 1901 | United Kingdom |
| 3 | ERR3577350 | 5e43defaf569ef0b47a5bf66 | 2013 | 225 | 1901 | United Kingdom |
| 4 | ERR234438 | 5e4bb0cac09ca059f1f8eba5 | 2004 | 225 | 1901 | United Kingdom |
| 5 | ERR234432 | 5e4bb0cac09ca021ddf8ebab | 2004 | 225 | 1901 | United Kingdom |
| 6 | SRR2736164 | 5e4ac02bc09ca0a93ef8e8fc | 2009 | 225 | 1901 | Canada |
| 7 | ERR234405 | 5e4bb0cac09ca0e837f8ebbe | 2004 | 225 | 1901 | United Kingdom |
| 8 | SRR3357190 | 5e455c5f66f40c483bb90c98 | 2011 | 225 | 1901 | United Kingdom |
| 9 | SRR3349937 | 5e45552706fc9417a7e7b3f4 | 2011 | 225 | 1901 | United Kingdom |
| 10 | ERR234495 | 5e4bb0cac09ca0510bf8eb73 | 2004 | 225 | 1901 | United Kingdom |
| 11 | ERR234434 | 5e4bb0cac09ca0671af8eba9 | 2004 | 225 | 1901 | United Kingdom |
| 12 | ERR234487 | 5e4bb0cac09ca02d5cf8eb79 | 2004 | 225 | 1901 | United Kingdom |
| 13 | SRR1661191 | 5e4abb4d5267942290016672 | 2006 | 225 | 1901 | Canada |
| 14 | ERR234466 | 5e4bb0cac09ca0acbff8eb8c | 2004 | 225 | 1901 | United Kingdom |
| 15 | ECDC_GR13055 | 5a27f1d4af9fa00001c3f490 | 2013 | 225 | 1901 | Greece |
| 16 | SRR3361336 | 5e468455c09ca08795f8dc5f | 2007 | 225 | 1901 | United Kingdom |
| 17 | ERR855166 | 5e57aa818bb687f08c0e332b | 2005 | 225 | 1901 | USA |
| 18 | SRR1661185 | 5e4abb4d52679400cd016677 | 2006 | 225 | 1901 | Canada |
| 19 | ERR234506 | 5e4bb0cac09ca0031af8eb6a | 2004 | 225 | 1901 | United Kingdom |
| 20 | SRR3343486 | 5e454db966f40c53e5b90718 | 2006 | 225 | 1901 | United Kingdom |
| 21 | ERR234496 | 5e4bb0cac09ca0c231f8eb72 | 2004 | 225 | 1901 | United Kingdom |
| 22 | ECDC_AT13_499 | 5a27f1e154e0d10001e0d11d | 2013 | 225 | 1901 | Austria |
| 23 | SRR3360813 | 5e455c5f66f40c4b58b90ddb | 2005 | 225 | 1901 | United Kingdom |
| 24 | ECDC_HU13_093 | 5a27f1b4af9fa00001c3f438 | 2013 | 225 | 1901 | Hungary |
| 25 | ERR222905 | 5e57a710f45f42144e15df75 | 2009 | 225 | 1901 | USA |
| 26 | ERR854944 | 5e57aa818bb687bff80e33f5 | 2013 | 1407 | 1901 | USA |
| 27 | ERR855400 | 5e57aa818bb68754d70e3259 | 2013 | 1407 | 7360 | USA |
| 28 | ERR191770 | 5e57a710f45f42641215df36 | 2009 | 1407 | 1901 | USA |
| 29 | ERR855295 | 5e57a70ff45f425e0315deab | 2010 | 1407 | 1901 | USA |
| 30 | ERR956685 | 5e57aa818bb68708800e3228 | 2013 | 1407 | 7360 | USA |
| 31 | ERR223620 | 5e57a70ff45f42043015def8 | 2009 | 1407 | 1901 | USA |
| 32 | ERR223623 | 5e57a70ff45f42758e15def5 | 2010 | 1407 | 10312 | USA |
| 33 | ERR2631844 | 5e4d1e6ec09ca028a5f8f79f | 2012 | 1407 | 1901 | USA |
| 34 | ERR855401 | 5e57aa818bb68717800e3258 | 2013 | 1407 | 1901 | USA |
| 35 | ERR3578957 | 5e43f1ccf569ef725aa5c0e8 | 2014 | 1407 | 1901 | United Kingdom |
| 36 | ERR1426748 | 5e4c0059526794bfdd016fa7 | 2015 | 1407 | 8143 | New Zealand |
| 37 | ERR854938 | 5e57aa818bb6879c7f0e33fb | 2012 | 1407 | 1901 | USA |
| 38 | ERR1426713 | 5e4c00595267940c1c016fc8 | 2010 | 1407 | 1901 | Australia |
| 39 | ERR223621 | 5e57a70ff45f4258a515def7 | 2010 | 1407 | 1901 | USA |
| 40 | ERR223630 | 5e57a70ff45f42795515def0 | 2010 | 1407 | 1901 | USA |
| 41 | ERR222894 | 5e57a710f45f42ed0915df7f | 2010 | 1407 | 1901 | USA |
| 42 | ERR3326126 | 5e7e1494c135817bb47f9a8e | 2017 | 1407 | 1901 | Norway |
| 43 | ECDC_PT13_065 | 5a27f25f890cc2000170ef07 | 2013 | 1407 | 1901 | Portugal |
| 44 | ECDC_DK13_482 | 5a27f1e2af9fa00001c3f4b6 | 2013 | 1407 | 1901 | Denmark |
| 45 | ERR855286 | 5e57aa818bb6871f6a0e32c1 | 2012 | 1407 | 1901 | USA |
| 46 | ECDC_T2_ES002 | 5a27f26a890cc2000170ef35 | 2013 | 1407 | 1901 | Spain |
| 47 | ERR191805 | 5e57a70ff45f420ccb15df18 | 2009 | 1407 | 1901 | USA |
| 48 | ERR971378 | 5e57aa818bb68786180e321d | 2011 | 1407 | 10312 | USA |
| 49 | ERR3325471 | 5e7e1e4e719b1a0c5633d9e7 | 2016 | 1407 | 1901 | Norway |
| 50 | ECDC_T2_ES011 | 5a27f2a854e0d10001e0d37d | 2013 | 1407 | 1901 | Spain |
| 51 | ECDC_BE13_447 | 5a27f1f4af9fa00001c3f4fe | 2013 | 2400 | 7363 | Belgium |
| 52 | ECDC_GC_072 | 5a27f1a7af9fa00001c3f41c | 2013 | 2400 | 7363 | Norway |
| 53 | SRR8559853 | 5e46ab90c09ca034b9f8deed | 2017 | 2400 | 7363 | Australia |
| 54 | SRR3349898 | 5e45552706fc94de3fe7b3ce | 2011 | 2400 | 7363 | United Kingdom |
| 55 | SRR3349669 | 5e45552706fc943179e7b344 | 2013 | 2400 | 7363 | United Kingdom |
| 56 | SRR8071135 | 5e5928e86530ab0db24f0661 | 2015 | 2400 | 7363 | USA |
| 57 | SRR3357168 | 5e455c5f66f40c1168b90c83 | 2015 | 2400 | 7363 | United Kingdom |
| 58 | ERR3579062 | 5e43f1ccf569ef573da5c151 | 2014 | 2400 | 7363 | United Kingdom |
| 59 | SRR8567021 | 5e46c134a233eccda0d062aa | 2017 | 2400 | 7363 | Australia |
| 60 | ERR2631932 | 5e4d1e6ec09ca0895df8f7ee | 2013 | 2400 | 7363 | USA |
| 61 | SRR3349290 | 5e454db966f40c1af1b90852 | 2013 | 2400 | 7363 | United Kingdom |
| 62 | ERR3201354 | 5e4d16d052679438390178e0 | 2013 | 2400 | 7363 | USA |
| 63 | SRR5827071 | 5e442a62f569ef36d9a5c649 | 2015 | 2400 | 7363 | New Zealand |
| 64 | ERR3201297 | 5e4d16d0526794c0c8017919 | 2012 | 2400 | 7363 | USA |
| 65 | SRR8071034 | 5e5928e96530ab535e4f079a | 2015 | 2400 | 1600 | USA |
| 66 | ERR855248 | 5e57aa818bb6875eea0e32e6 | 2011 | 2400 | 7363 | USA |
| 67 | SRR5827265 | 5e442a62f569ef488ca5c6ff | 2015 | 2400 | 7363 | New Zealand |
| 68 | SRR3343566 | 5e454db966f40c1733b90761 | 2013 | 2400 | 7363 | United Kingdom |
| 69 | SRR3349904 | 5e45552706fc94ae36e7b3d4 | 2011 | 2400 | 7363 | United Kingdom |
| 70 | ECDC_T2_ES039 | 5a27f2ae54e0d10001e0d391 | 2013 | 2400 | 7363 | Spain |
| 71 | SRR3349624 | 5e454db966f40c24b4b908ed | 2013 | 2400 | 7363 | United Kingdom |
| 72 | ERR3578988 | 5e43f1ccf569ef6993a5c107 | 2014 | 2400 | 7363 | United Kingdom |
| 73 | SRR8559538 | 5e46a3b3b2c89995028c97f3 | 2017 | 2400 | 7363 | Australia |
| 74 | SRR3357356 | 5e455c5f66f40cac71b90ced | 2015 | 2400 | 7363 | United Kingdom |
| 75 | SRR5827156 | 5e442a62f569eff7f8a5c699 | 2014 | 2400 | 7363 | New Zealand |
| 76 | ERS135301 | 58ac20eec492e60001aa1274 | 2010 | 2992 | 9363 | USA |
| 77 | SRR3361164 | 5e468455c09ca03a9ef8dbd1 | 2012 | 2992 | 9363 | United Kingdom |
| 78 | SRR3343481 | 5e454db966f40c0082b90713 | 2013 | 2992 | 9363 | United Kingdom |
| 79 | ECDC_IT2926 | 5a27f199af9fa00001c3f408 | 2013 | 2992 | 9363 | Italy |
| 80 | SRR3360746 | 5e455c5f66f40ca81bb90dc2 | 2010 | 2992 | 11463 | United Kingdom |
| 81 | SRR3349636 | 5e454db966f40c27ceb908f8 | 2014 | 2992 | 11864 | United Kingdom |
| 82 | SRR8560581 | 5e46b483b2c89961708c9b5e | 2017 | 2992 | 11864 | Australia |
| 83 | ERR3577398 | 5e43e77df01281756fce72eb | 2014 | 2992 | 11864 | United Kingdom |
| 84 | SRR3349841 | 5e45552706fc94922ee7b39b | 2012 | 2992 | 9363 | United Kingdom |
| 85 | ECDC_NL28 | 5a27f1d6890cc2000170ed8d | 2013 | 2992 | 9362 | Netherlands |
| 86 | ERR191767 | 5e57a710f45f425fa415df38 | 2009 | 2992 | 9363 | USA |
| 87 | SRR3349815 | 5e45552706fc942370e7b382 | 2011 | 2992 | 11463 | United Kingdom |
| 88 | SRR5990383 | 5e5928e96530ab821c4f06de | 2015 | 2992 | 13145 | USA |
| 89 | ERR855305 | 5e57aa818bb6876b110e32b0 | 2011 | 2992 | 9363 | USA |
| 90 | SRR3349557 | 5e454db966f40c5188b908b2 | 2014 | 2992 | 9362 | United Kingdom |
| 91 | SRR3343598 | 5e454db966f40ca722b9077f | 2013 | 2992 | 9363 | United Kingdom |
| 92 | ERR2631800 | 5e4d1e6ec09ca0edaef8f77f | 2013 | 2992 | 9363 | USA |
| 93 | SRR5827266 | 5e442a62f569ef5f9fa5c700 | 2015 | 2992 | 9363 | New Zealand |
| 94 | ECDC_IT2933 | 5a27f1e854e0d10001e0d139 | 2013 | 2992 | 9363 | Italy |
| 95 | SRR8567262 | 5e46c134a233ec6d3cd0639b | 2017 | 2992 | 11428 | Australia |
| 96 | SRR3349825 | 5e45552706fc949444e7b38b | 2011 | 2992 | 11463 | United Kingdom |
| 97 | SRR3343671 | 5e454db966f40c35bab907ba | 2013 | 2992 | 11463 | United Kingdom |
| 98 | SRR8560304 | 5e46af28c09ca0d033f8e1a3 | 2017 | 2992 | 11428 | Australia |
| 99 | SRR3361158 | 5e468455c09ca0869bf8dbcd | 2012 | 2992 | 11428 | United Kingdom |
| 100 | SRR8566491 | 5e46b483b2c89928ec8c9b75 | 2017 | 2992 | 9362 | Australia |
| 101 | SRR8566641 | 5e46b84dc09ca0292df8e422 | 2017 | 4186 | 7359 | Australia |
| 102 | SRR8566720 | 5e46b84dc09ca0259af8e471 | 2017 | 4186 | 7359 | Australia |
| 103 | SRR8559463 | 5e46a3b3b2c8999cb78c97a8 | 2017 | 4186 | 7359 | Australia |
| 104 | SRR8559954 | 5e46ab90c09ca0c847f8df52 | 2017 | 4186 | 7359 | Australia |
| 105 | SRR8559626 | 5e46a3b3b2c8997d8e8c984b | 2017 | 4186 | 7359 | Australia |
| 106 | SRR8559546 | 5e46a3b3b2c899f9378c97fb | 2017 | 4186 | 7359 | Australia |
| 107 | ERR3325297 | 5e7e1e4e719b1ae95533da7a | 2016 | 4186 | 7359 | Norway |
| 108 | SRR8560017 | 5e46ab90c09ca08510f8df66 | 2017 | 4186 | 7359 | Australia |
| 109 | SRR8566960 | 5e46c134a233ec5665d0626d | 2017 | 4186 | 7359 | Australia |
| 110 | ERR3578058 | 5e43ebd2f012811f7ece75ef | 2016 | 4186 | 7359 | United Kingdom |
| 111 | SRR8566949 | 5e46c134a233ec2d36d06262 | 2017 | 4186 | 7359 | Australia |
| 112 | SRR8560383 | 5e46b483b2c89918798c9a98 | 2017 | 4186 | 7359 | Australia |
| 113 | ERR3325861 | 5e7e1494c13581192f7f99c7 | 2017 | 4186 | n/d | Norway |
| 114 | SRR5827207 | 5e442a62f569ef03cea5c6c7 | 2014 | 4186 | 7359 | New Zealand |
| 115 | SRR8566835 | 5e46b84dc09ca06481f8e4e4 | 2017 | 4186 | 7359 | Australia |
| 116 | SRR5801828 | 5e4bed08c09ca02796f8ee5c | 2014 | 4186 | 7359 | Australia |
| 117 | SRR8560402 | 5e46b483b2c899deac8c9aab | 2017 | 4186 | 7359 | Australia |
| 118 | SRR8560491 | 5e46b483b2c8997a7d8c9b04 | 2017 | 4186 | 7359 | Australia |
| 119 | ERR3325292 | 5e7e1e4e719b1a2dce33da7e | 2016 | 4186 | 7359 | Norway |
| 120 | SRR5801862 | 5e4bed08c09ca04f85f8ee3b | 2012 | 4186 | 7359 | Australia |
| 121 | SRR8560106 | 5e46af28c09ca0d7f9f8e0de | 2017 | 4186 | 7359 | Australia |
| 122 | SRR8560450 | 5e46b483b2c89924708c9adb | 2017 | 4186 | 7359 | Australia |
| 123 | SRR5801825 | 5e4bed08c09ca0855ef8ee5f | 2014 | 4186 | 7359 | Australia |
| 124 | SRR5801827 | 5e4bed08c09ca034c1f8ee5d | 2014 | 4186 | 7359 | Australia |
| 125 | SRR5801849 | 5e4bed08c09ca0c61ff8ee48 | 2012 | 4186 | 7359 | Australia |
